# Temporal regulation of HTLV-2 expression in infected cell lines and patients: evidence for distinct expression kinetics with nuclear accumulation of APH-2 mRNA

**DOI:** 10.1186/1742-4690-9-74

**Published:** 2012-09-13

**Authors:** Cecilia Bender, Francesca Rende, Alessia Cotena, Paola Righi, Paola Ronzi, Ilaria Cavallari, Claudio Casoli, Vincenzo Ciminale, Umberto Bertazzoni

**Affiliations:** 1 Department of Life and Reproduction Sciences, Section of Biology and Genetics, University of Verona, Verona, Italy; 2 Department of Surgery Oncology and Gastroenterology, University of Padova, Padua, Italy; 3 Department of Clinical Sciences, University of Milan, Milan, Italy; 4 Istituto Oncologico Veneto IRCCS, Padua, Italy; 5 Gemiblab, Vicolo Asse 1 43121, Parma, Italy

## Abstract

**Background:**

Human T-cell leukemia virus types 1 and 2 (HTLV-1 and HTLV-2) are delta retroviruses with similar genetic organization. Although both viruses immortalize T-cells *in vitro*, they exhibit distinct pathogenic potential *in vivo*. To search for possible differences in its expression strategy with respect to HTLV-1, we investigated the pattern of HTLV-2 expression in infected cell lines and peripheral blood mononuclear cells (PBMCs) from infected patients using splice site-specific quantitative RT-PCR.

**Findings:**

A novel alternative splice acceptor site for exon 2 was identified; its usage in *env* transcripts was found to be subtype-specific. Time-course analysis revealed a two-phase expression kinetics in an infected cell line and in PBMCs of two of the three patients examined; this pattern was reminiscent of HTLV-1. In addition, the minus-strand *APH2* transcript was mainly detected in the nucleus, a feature that was similar to its HTLV-1 orthologue *HBZ*. In contrast to HTLV-1, expression of the mRNA encoding the main regulatory proteins Tax and Rex and that of the mRNAs encoding the p28 and truncated Rex inhibitors is skewed towards p28/truncated Rex inhibitors in HTLV-2.

**Conclusion:**

Our data suggest a general converging pattern of expression of HTLV-2 and HTLV-1 and highlight peculiar differences in the expression of regulatory proteins that might influence the pathobiology of these viruses.

## Findings

Human T-cell leukemia virus types 1 and 2 (HTLV-1 and HTLV-2) are related deltaretroviruses [1] with similar genetic organization and expression strategies [2-4]. Both viruses immortalize T-cells in culture and establish a persistent infection *in vivo *[5]. However, unlike HTLV-1, which causes adult T-cell leukemia/lymphoma (ATLL) and tropical spastic paraparesis/HTLV-1-associated myelopathy (TSP/HAM), HTLV-2 has not been linked to lymphoproliferative diseases although an increase in lymphocytes counts was described in HTLV-2-infected patients [6] and coinfection with HTLV-2 plays an important role in the progression of HIV-infected patients to AIDS [7].

**Figure 1:**
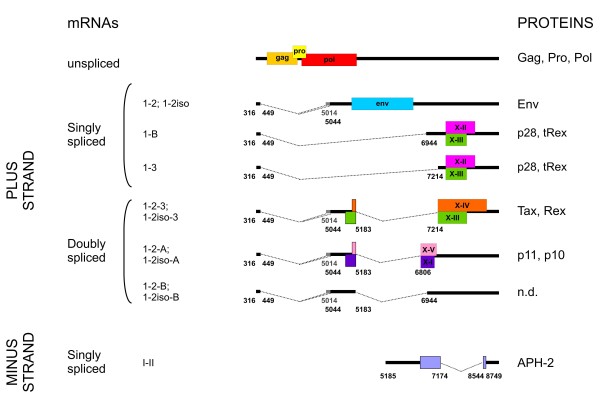
**Genetic organization, alternative splicing and coding potential of HTLV-2 mRNAs.** Structure and coding potential of HTLV-2 alternatively spliced mRNAs. ORFs are indicated by boxes. Splice sites are indicated by numbers and correspond to the Mo-T isolate (GeneBank: M10060) in which + 1 is the first base of U3 in the 5’ LTR. Gray lines and numbers designate the 5’ end of exon 2iso identified in the present study. n.d.: the protein coding potential of this transcript has not yet been determined.

Like HTLV-1, HTLV-2 produces plus- and minus-strand alternatively spliced transcripts that code for virion components and non-structural proteins, including Tax and Rex that regulate viral expression at the transcriptional and post-transcriptional levels, respectively [8,9]. In contrast to HTLV-1, the kinetics of expression of the individual HTLV-2 transcripts has not been described so far. To address this point, we employed splice site-specific quantitative RT-PCR (qRT-PCR) to measure individual HTLV-2 mRNAs (Figure 1) and their expression kinetics in chronically infected cell lines and in peripheral blood mononuclear cells (PBMCs) obtained from HTLV-2-infected individuals. Results revealed interesting analogies and differences from HTLV-1 [10].

### Identification of a novel HTLV-2 splice acceptor-site

**Figure 2:**
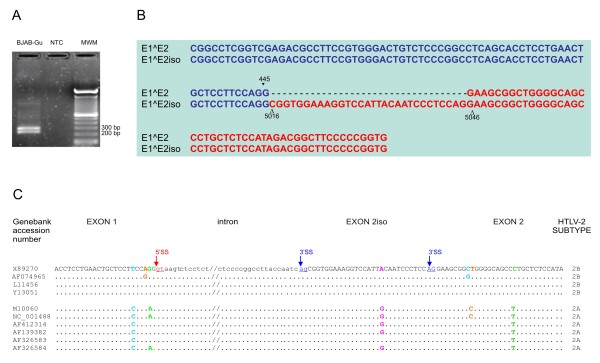
** Identification of a novel HTLV-2 splice acceptor-site.A**. Agarose gel electrophoresis of exon 1–2 and exon 1-2iso RT-PCR products. BJAB-Gu cDNA was amplified using the 6000-Gu forward primer (5’ GGCGGGCTCCTTCAACG 3’) located in the first exon and the 5183-Gu reverse primer (5’ CCATGGTGTTGGTGGTCT 3’) in the second exon. Amplification yielded the expected 275-bp band and a slightly longer product (lane 1). No bands were detected in the no template control (NTC, lane 2); lane 3 shows a 1-kbp molecular weight marker (MWM). Cell lines were cultured at 37°C, 5% CO_2_, in RPMI 1640 supplemented with 10% fetal bovine serum, 2 mM L-glutamine, penicillin (100 U/ml) and streptomycin (100 μg/ml). Cells were lysed in TRIzol® (Invitrogen) and total RNA was extracted and processed as detailed in Additional file 1: Table S1. **B**. Sequence alignment of cloned PCR products and identification of a novel splice acceptor site upstream of exon 2. Nucleotides in exon 1 are in blue and nucleotides in exon 2 are in red. Sequence analysis demonstrated that the 1-2iso junction is generated by splicing to a novel 3’splice-site (SS) located 30 nucleotides upstream of the exon 2 3'SS. Numbering of the sequence corresponds to that of the Gu isolate (GenBank: × 89270.1) in which + 1 is the first base of U3 in the 5’ LTR. ▼ indicates nucleotide position in exon 1; Δ indicates nucleotide position in exon 2 or 2iso. **C**. Sequence alignment of HTLV-2 isolates. DNA sequences containing exon 1 splice donor (5’SS) and exon 2iso or exon 2 splice-acceptor (3’SS) sites were compared between various HTLV-2 isolates retrieved from GenBank (accession numbers are indicated on the left, subtypes on the right). Exons and introns are indicated in upper and lower case, respectively. Underlined nucleotides indicate the 5’SS and 3’SS consensus sequences. The consensus sequence AGgtaagt at the exon 1-intron boundary varies among 2A and 2B subtypes, while the intronic gt 5’SS and ag 3’SS dinucleotides are invariable. The 3'SS defining exon 2iso is conserved among the different isolates examined.

We first analyzed the pattern of viral mRNAs in the HTLV-2-infected cell lines Mo-T (infected with subtype A) and BJAB-Gu (infected with subtype B). Pilot experiments showed unexpectedly low (almost 3 orders of magnitude) levels of the *env* mRNA in BJAB-Gu cells compared to Mo-T. Further experiments in the BJAB-Gu cell line using a sense primer located at the 5’ end of exon 1 and an antisense primer located in exon 2 yielded the expected 275 bp band and a slightly longer product (Figure 2A). Sequence analysis of cloned PCR products indicated that the longer product was generated by usage of a novel splice acceptor site (termed "*2iso*") located 30 nucleotides upstream of exon 2 (Figure 2B). *In silico* analysis of different HTLV-2 isolates showed that the novel 2iso 3’splice-site (SS) defines a *GU-AG* type intron with a consensus “*AGgtaagt*” sequence at the exon 1-intron boundary that varied among the isolates (Figure 2).

### Quantitative and temporal analysis of HTLV-2 mRNAs in infected cell lines

**Figure 3:**
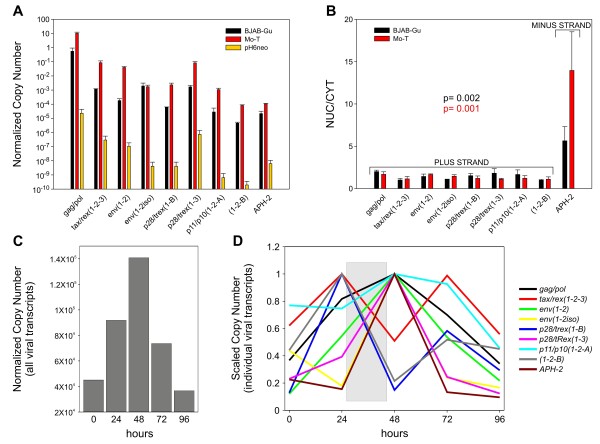
**Quantitative analysis, intracellular compartmentalization and expression kinetics of HTLV-2 mRNAs in infected cell lines.** Normalized copy number (NCN) and Scaled Copy Numbers (SCN) were calculated as described in the text. Housekeeping genes used for normalization were GAPDH (panels **A** and **B**) and 18S rRNA (panels **C** and **D**) and in this latter case (panel **C**) the obtained NCN were multiplied for 10^7^. **A**. NCN of viral mRNAs in the HTLV-2-infected cell lines BJAB-Gu (black bars) and Mo-T (red bars) and in cells transfected with the HTLV-2A molecular clone pH6neo (yellow bars). Cells were harvested at densities ranging from 100,000/ml (transfected cells) to 500,000/ml (infected cell lines). Shown are mean values of 3 independent experiments and standard error bars. **B**. Nucleo-cytoplasmic distribution (NUC/CYT) of plus- and minus-strand HTLV-2 mRNAs in infected cell lines Mo-T (red bars) and BJAB-Gu (black bars) was determined as detailed in Additional file 1: Table S1. Shown are mean values of 4 independent experiments, standard error bars and P values. The square brackets in the figure indicate a statistically significant difference between plus-and minus-strand transcripts calculated with a Mann–Whitney Rank Sum Test (p = 0.001 and p = 0.002 for the Mo-T and BJAB-Gu cell lines respectively). **C**,**D**. Temporal analysis of HTLV-2 expression. A confluent culture of BJAB-Gu cells was diluted to 25,000 cells/ml and aliquots were harvested every 24 h over a 96-h period. Shown are NCN of the sum of all (plus and minus strand) viral transcripts at each time point (**C**) and SCN of individual mRNAs (**D**). The grey box in the figure (panel **D**), highlights the switch in the pattern of expression from early to late transcripts.

**Figure 4:**
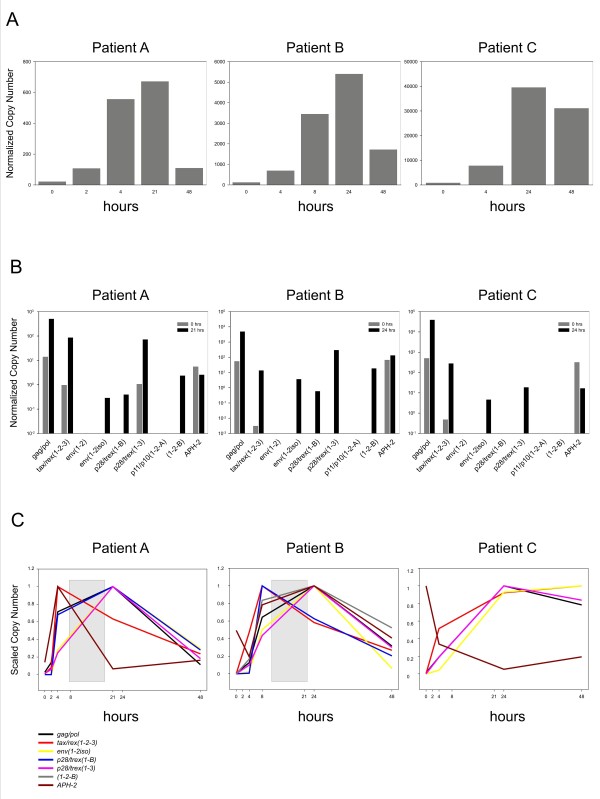
**Temporal analysis of HTLV-2 expression in PBMCs from infected patients.** Normalized copy number (NCN) and Scaled Copy Numbers (SCN) were calculated as described in the text. The housekeeping gene used for the normalization was 18S rRNA and the resulted NCN was multiplied for 10^7^. **A**. NCN of the sum of all viral mRNAs (plus- and minus- strands) viral mRNAs in PBMCs from infected patients in a 48-h time course of culture *in vitro*. PBMCs were cultured and harvested for total RNA at the indicated time points. **B**. NCN of the individual mRNAs in HTLV-2-infected patients at time 0 (grey bars) and after 21 (patient A) or 24 (patients B and C) hours of culture (black bars). **C**. SCN of individual mRNAs are plotted over a 48-h time period. The grey box in the figure, highlights the switch in the pattern of expression from early to late transcripts.

We next tested the expression levels of the different HTLV-2 mRNAs using splice site-specific primer sets and qRT-PCR (Additional file 1: Table S1), and we calculated the Normalized Copy Number (NCN) by dividing the absolute copy number of each transcript by the absolute copy number of the housekeeping gene (GAPDH or 18S rRNA, see Figures 3 and 4).

Using two splice site-specific primer pairs, we quantified the levels of the alternative *env* transcripts [*env*(*1*–*2)* and *env(1-2iso)*] from the Mo-T and BJAB-Gu cell lines and in cells transfected with the HTLV-2A molecular clone pH6neo (kindly provided by Dr. P. L. Green). Results showed that the *env(1-2iso)* mRNA was expressed at similar levels in both cell lines, while the canonic *env(1–2)* transcript was about 100-fold less abundant in BJAB-Gu cells (Figure 3A). Interestingly, the HTLV-2A molecular clone pH6neo also expressed mainly *env(1–2)*, suggesting a subtype-specific usage of the 2 vs. 2iso 3’SS (Figure 3A). While the sequence of the acceptor and donor dinucleotides is conserved between the two isolates, the adjacent nucleotides are different (Figure 2C), suggesting that this could result in a different splice site usage in the different isolates. The 1-2iso splice acceptor site was also used in the context of a doubly spliced *tax/rex(1-2iso-3)* mRNA which was readily detected in both cell lines and in the molecular clone pH6neo (data not shown). Selection of 1-2iso 3'SS as opposed to the 1–2 3'SS is not predicted to affect the coding potential of the *env(1–2; 1-2iso)*, *tax/rex(1-2-3; 1-2iso-3)* or *1-2-B* and *1-2iso-B* mRNAs, as both 3'SS are located in the non-coding portion of the transcripts. Interestingly, the 1-2iso splice site is not unique to only the BJAB-Gu cell line, as it is efficiently used in the patients, which are infected with subtype 2B (see next paragraph).

In the chronically infected cell lines Mo-T and BJAB-Gu as well as in cells transfected with the pH6neo molecular clone the most abundant viral mRNA was *gag/pol* followed by *tax*/*rex(1-2-3), env* and *p28/trex*(*13)* (Figure 3A); the latter codes for p28 and the truncated isoforms of Rex (p22/20 and p19/p18). We proposed to term these truncated isoforms of Rex as “tRex” [11]. Both p28 and tRex may act as latency factors by inhibiting the *tax/rex* mRNA [12] and the Rex protein, respectively [13]. Interestingly, in HTLV-2 *tax/rex* was detected at levels comparable to *p28/trextaxrex/trex(1–3)* ratio = 0.5 in pH6neo-transfections; 1.04 in Mo-T; 0.69 in BJAB-Gu]. This is in contrast with HTLV-1, where *tax/rex* is much more abundant than the mRNAs coding for p30Tof and p21Rex, the orthologs of p28 and tRex, respectively [10]. These data suggest that the ratio of expression of the *tax/rex* mRNA vs. the mRNAs encoding potential inhibitors is higher in HTLV-1[10] compared to HTLV-2. The p28 and tRex proteins are also coded by the less abundant *p28/trex(1-B)* mRNA. Transcript 1-2-B, whose protein product has not yet been identified and is also expressed as *1-2iso-B;* the *p11/p10(1-2-A)* transcript and the minus-strand *APH-2* transcript were all expressed at lower levels. These data are consistent with a previous study by Li et al. [14].

A recent study on the intracellular distribution of HTLV-1 mRNAs indicated that the plus-strand transcripts showed similar partition in the nucleus and cytoplasm, while the minus-strand mRNAs were over 10-fold more abundant in the nucleus, suggesting that they may play a role as a non-coding RNAs [10]. To test whether this is the case for HTLV-2, we analyzed the nucleo-cytoplasmic distribution of plus- and minus-strand HTLV-2 mRNAs. Results obtained in the Mo-T and BJAB-Gu infected cell lines showed that all the plus-strand mRNAs were equally distributed in the nucleus and cytoplasm, while the minus-strand *APH-2* mRNA [15] was about 10-fold or 4-fold higher (Mo-T and BJAB-Gu cell lines, respectively) in the nucleus (Figure 3B; Mann–Whitney Rank Sum Test; p = 0.001 and p = 0.002 for the Mo-T and BJAB-Gu cell lines respectively). This finding is reminiscent of cell lines chronically infected with HTLV-1 [10].

We next investigated whether, in analogy to HTLV-1 [11], HTLV-2 is characterized by an early/late temporal pattern of expression. To test this possibility in an *in vitro* model, we induced HTLV-2 expression in BJAB-Gu cells by diluting to 25,000 cells/ml starting from a confluent culture and measured transcript levels over 96 h by qRT-PCR. As indicated in Figure 3C, this treatment results in an overall upregulation of viral expression. To ensure that the pattern of viral expression was not affected by cell death, we tested cell viability by trypan blue exclusion at each time point and found that cell death did not exceed 4% for up to 96 h of culture. The amount of p19 released by the BJAB-Gu cells was below the threshold of detection by ELISA at 0 and 24 h and rose to 62 pg/ml at 48 h, 121 pg/ml at 72 h, and 169 pg/ml at 96 h.

To better visualize the timing of expression of individual mRNAs, we calculated the scaled copy number (SCN) by dividing the normalized copy number (NCN) of each transcript at each time point by the maximum NCN measured for that mRNA during the experiment. Results showed 2-phase kinetics with the expression of *tax/rex(1-2-3)*, *p28/tRex(1-B)* and *1-2-B* preceding that of *gag/pol*, *env* and *p28/trex(1–3)* mRNAs (Figure 3D), a finding that is consistent with the key role of Tax and Rex as master regulatory proteins driving expression of the other viral genes.

### Temporal analysis of HTLV-2 expression in PBMCs from infected patients

We next used qRT-PCR to investigate HTLV-2 expression kinetics in PBMCs obtained from 3 HTLV-2B-infected patients; these patients had similar proviral loads and were HTLV-1- and HIV-1-negative (Additional file 2: Table S2). All of the recruited patients gave informed consent, according to the Italian laws and the Declaration of Helsinki. The study was approved by the institutional ethics committees of all participating institutions. As described for HTLV-1 [10], a sharp upregulation of HTLV-2 transcripts was observed upon culture of the PBMCs in all the examined patients (Figure 4A). Before *in vitro* culture only *gag/pol*, *APH2* and *tax/rex(1-2-3)* were detected in all patients; *p28/trex*(*13*) was detected only in patient A (Figure 4B, grey bars). Upon *in vitro* culture the expression of all transcripts was sharply upregulated (Figure 4B, black bars); however, patient C exhibited a marked downregulation of *APH-2.* Following reactivation, *gag/pol* was the most abundant transcript. *Tax/rex(1-2-3), p28/trex*(*13*), *APH-2*, and in patients A and B, *1-2-B* were also readily detected, while the other transcripts were expressed at much lower levels. Consistent with the fact that the patients were infected with HTLV-2 subtype B, *env(1-2iso)* was efficiently detected, while *env(1–2)* was undetectable. Patient C did not express *p28/trex(1-B)* or (*1-2-B)*. *p11/p10(1-2-A)* was below the threshold of detection in all patients. The *tax/rex over p28/trex(1–3)* ratio ranged between 0.9 and 7.6 (depending on the time point) in patient A, and was even more skewed in favor of *p28/trex(1–3)* in patient B (ratios between 0.04 and 0.37, depending on the time point).

SCN calculations (Figure 4C) indicated a two-phase kinetics of HTLV-2 gene expression in patients A and B, with an early sharp rise in *tax/rex(1-2-3)* expression followed by expression of all other genes. Patient B also expressed *p28/trex(1-B)* as an early transcript, although it its expression levels were lower by about 3 orders of magnitude compared to the *p28/tRex(1–3)* (Figure 4B).

The expression pattern of patients A and B is similar to that of the BJAB-Gu cell line (Figure 3D) and suggests an early/late switch in the pattern of viral expression. While plus-strand transcripts showed an overall common pattern of expression in two patients and in the BJAB-Gu cell line, the temporal expression of *APH-2* appeared to be highly variable, at least in the restricted number of samples examined. Further studies carried out on larger cohorts of samples should be performed, to test the possible correlation of different *APH-2* expression patterns with virus load and patient status. In this regard, a recent study showed that both Tax and *APH-2* mRNA levels correlate with proviral load; interestingly, no correlation was found between *APH-2* levels and the number of lymphocytes in the patients, suggesting that, in contrast to HBZ, APH-2 does not promote cell proliferation [16].

Although the temporal pattern of HTLV-2 expression was reminiscent of that of HTLV-1, it is noteworthy that the most abundant HTLV-2 mRNA encoding p28 and tRex (i.e. *mRNA1-3*) was expressed from a “late” transcript, while HTLV-1 p21Rex was expressed as an early transcript in most patients examined in a previous study [10].

Taken together with previous analyses of HTLV-1 expression, the present study suggests a converging expression strategy of the two viruses characterized by 2-phase expression kinetics of viral mRNAs and by marked nuclear localization of minus-strand transcripts. Our results also highlight the following differences between the 2 viruses: (i) the relative expression of the *tax/rex* mRNA compared to the mRNAs coding for potential inhibitors of Tax (*p30Tof and p28*) and Rex [(*p21Rex and p28/tRex(1–3*)] is skewed toward *tax/rex* in HTLV-1 and towards the inhibitors in HTLV-2; (ii) the *p28/trex* mRNA was expressed as a “late” transcript in HTLV-2 while mRNAs for HTLV-1 orthologs *p30/Tof* and *p21Rex* are expressed early in most patients examined, suggesting interesting differences in the fine-tuning of Rex function between the two viruses. Nevertheless, it is appropriate to be cautious in the interpretation of these data, since only three patients were examined. This limitation was due to the fact that the kinetics analysis required (i) large numbers of PBMC, (ii) a high proviral load, and (iii) no coinfection with HIV or HTLV-1, which severely limited the number of samples suitable for this analysis.

Future studies should be aimed at further investigating the functions of these regulatory proteins in HTLV-1 and HTLV-2 and at understanding how they may contribute to the different pathobiology of the two viruses.

## Competing interests

The authors declare that they have no competing interests.

## Authors’ contributions

CB identified the novel splice acceptor site and carried out kinetics of expression in cell lines and patients. FR carried out nucleo-cytoplasmic fractionations and real-time RT-PCR assays; AC and PR set real-time RT-PCR and measured mRNA expression in cell lines; PR carried out cell lines and patients cultures; IC carried out cell culture and transfection; CC provided patient samples and supervised cell cultures; VC and UB designed the experiments and prepared the manuscript; and all authors contributed to the analysis and interpretation of the data. All authors read and approved the final manuscript.

## Supplementary Material

Additional file 1 ** Table S1.** List of primer sets and probes used in qPCR detection of HTLV-2 proviral load or HTLV-2 mRNAs expression. Click here for file

Additional file 2 ** Table S2. **Clinical and immunological features of HTLV-2 infected patients. Click here for file
