# Supplementary material for: Temporal regulation of HTLV-2 expression in infected cell lines and patients: evidence for distinct expression kinetics with nuclear accumulation of APH-2 mRNA
Source: Retrovirology. 2012 Sep 13;9:74. doi: 10.1186/1742-4690-9-74 (PMC3468393; doi:10.1186/1742-4690-9-74)
Supplement: Additional file 1 — Table S1. List of primer sets and probes used in qPCR detection of HTLV-2 proviral load or HTLV-2 mRNAs expression. [file 1742-4690-9-74-S1.pdf]

Target	Primer	Primer Sequence	Working concentration (nM)	Probe
**gag/pol**	GP2-S*	GCCTACCCAAGCGCTACTT	300	TMP-8*
	GP2-AS*	CCCGGGCACGAGTGTCT	300	
**env (1^2)**	TR2-S*	TGCTCCTCCCAAG^GAAGC	600	TMP-6*
	ENV2-AS*	AGTAGGAAGAAAACATTACCCATGGT	600	
**env (1^2iso)**	E1^E2-2b-S	TGCTCCTTCCAGG^CGGTG	500	TMP-6*
	ENV2-AS	AGTAGGAAGAAAACATTACCCATGGT	300	
**tax/rex (2/2iso^3)**	Tax2b-S	ACCATGG^CCCATTCCCAAGG	300	TMP-7*
	P28-AS*	GGACACCAATCGGCCTGTAC	300	
**1-B**	P28-1TC-S	GCTCCTCCCAAG^GCGCTC	300	TMP-7*
	P28-AS	GGACACCAATCGGCCTGTAC	300	
**1-3**	P28-2-S*	TGCTCCTCCCAAG^CCCAT	300	TMP-7*
	P28-AS	GGACACCAATCGGCCTGTAC	300	
**1-2-A (2/2iso^A)**	EXON2-S	CTGCTCTCCATAGACGGC	300	TMP-6*
	P10-AS*	GGGAAAAGAAGGTC^CCATG	150	
**1-2-B (2/2iso^B)**	EXON2-S	CTGCTCTCCATAGACGGC	300	TMP-6*
	?-AS*	GCAGAAAGGAGCGC^CCAT	150	
**APH-2 (I^II)**	APH-2-S	GGATGGATCCCCAAG^ACTA	300	TMP-9*
	APH-2-AS	GCTGTCCTTCTTGGTTCCTC	300	
**pol**	pol-2-S	CCCCGTCGTGCTTGACA	200	pol-2 probe
	pol-2-AS	GGACGTAYGCTGCCTTTTGAG	200	
**Albumin**	alb-S	GCTGTCATCTCTTGTGGGCTGT	200	alb probe
	alb-AS	ACTCATGGGAGCTGCTGGTTC	200	

Table S1. List of primer sets and probes used in qPCR detection of HTLV-2 proviral load or HTLV-2 mRNAs expression.

Splice junction-specific primers were designed to span the exon-exon junction of the different mRNAs; ^ indicates the splice junction between two exons; asterisks indicate previously described primers and probes [*Li M and Green PL, J Virol Methods. 2007, 142:159-68*].

PBMCs were isolated from HTLV-2 carriers by gradient centrifugation with Lymphoprep® (Nycomed Pharma As). DNA was extracted using the GFXTM Genomic Blood Purification Kit (Amersham Biosciences) and its concentration was determined by spectrophotometry. Short-term cultures were performed by culturing PBMCs for two days in RPMI 1640 medium supplemented with 10% fetal calf serum, 2 mM L-glutamine and 20 U/mL IL-2. At different time points, 2-3 X 10^6 PBMCs or 1,5 X 10^6 BJAB-Gu and Mo-T chronically infected cell lines were harvested in TRIzol® (Invitrogen) for total RNA extraction. Nuclear and cytoplasmic RNA fractions were obtained from chronically infected cell lines using the Paris Kit (Ambion). Nuclear and cytoplasmic RNA was processed as previously described [*Rende F, et al., Blood 2011:4855-4859*]. RNA was quantitated using a NanoDrop™ 2000 spectrophotometer (Thermo Scientific) and its quality was assessed using an Agilent Bioanalyzer 2100 platform (Agilent). Aliquots of total RNA (1 μg) were treated with DNAse I enzyme (Invitrogen) and reverse transcribed with the QuantiTect Reverse Transcription kit (Qiagen) or SSII reverse transcriptase (Invitrogen).

PCR reactions were performed with a Mastercycler Realplex (Eppendorf) or an ABI Prism 7900 HT Sequence Detection System (Applied Biosystems). Quantitation of the *pol* gene for proviral load determination was normalized to that of the *albumin* gene. The pol-2 probe sequence was 5’FAM-CCCCCTGCCTTTTTTCCGA-TAMRA3’, the albumin probe was 5’JOE-CCTGTCATGCCCACACAAATCTCTCC-TAMRA3’. The final reaction volume was 15 μl, consisting of RealMasterMix Probe ROX (5 prime), probe (100 nM), primers (at the final concentration indicated in Table S1), and genomic DNA (100 ng).

Quantification of viral mRNAs was normalized against the human 18S rRNA (Figure 3C and D; 4A -C) or GAPDH mRNA (Figure 3A and B) (Taqman® Gene expression Master Mix, Applied Biosystems) and indicated as NCN (normalized copy number). Platinum® SYBR® Green (Invitrogen) (Figure 3A and B) or TaqMan probes (Figure 3C and D; Figure 4A-C) were used. The final reaction volume was 25 µl, consisting of cDNA (5 µl of each reverse transcription), primers (at the final concentration indicated in Table S1), Platinum® SYBR® Green or Universal PCR Master Mix (Applied Biosystems) and probes. The final probe concentration was 250 nM (TMP-9) or 200 nM (all other probes).

For each run, standard samples, DNA/cDNA samples and negative controls (no reverse transcription and no template controls) were assayed in triplicate. Standard curves, included within every plate, were generated by 10-fold serial dilutions of plasmids containing the different amplicons (Figure 3A, C and D; Figure 4 A-C) or cDNA from the Mo-T or BJAB-Gu cell lines (Figure 3B, red and black bars respectively), for HTLV-2 transcripts quantification, or genomic DNA from the C344 cell line for proviral load determination (indicated in Table S2). Cycling conditions were 94°C for 2 min, followed by 50 cycles at 94°C for 15 sec and 60°C for 1 min. Regression curve analysis, consisting of 5 points tested in triplicate, indicated an efficiency higher than 90% and a Spearmann's correlation coefficient ≥ 0.9. Detection cut off ranged from 10 to 50 copies before normalization. Data were not considered if the cycle thresholds (Cts) of the triplicates differed by more than 0.35, in which case the reaction was repeated.
