# Supplementary material for: Temporal regulation of HTLV-2 expression in infected cell lines and patients: evidence for distinct expression kinetics with nuclear accumulation of APH-2 mRNA
Source: Retrovirology. 2012 Sep 13;9:74. doi: 10.1186/1742-4690-9-74 (PMC3468393; doi:10.1186/1742-4690-9-74)
Supplement: Additional file 2 — Table S2. Clinical and immunological features of HTLV-2 infected patients. [file 1742-4690-9-74-S2.pdf]

Patient	Sex	Age	HIV-1	HTLV-1	HTLV-2 proviral load	HTLV-2 subtype
A	male	53	-	-	2566	B
B	male	54	-	-	5700	B
C	female	56	-	-	4627	B

Table S2. Clinical and immunological features of HTLV-2-infected patients.

All recruited individuals gave informed consent, according to Italian laws, and were enrolled as part of a cohort of drug users (IDUs) treated at Italian hospitals and therapeutic communities. Patients were tested for the presence of antibodies against HTLV according to the HTLV European Research Network (HERN) algorithm [*Taylor, G.P. Journal of Acquired Immune Deficiency Syndromes & Human Retrovirology, 1996 13:S8-S14*]. All patients were infected with HTLV-2 subtype B, as determined by nested PCR followed by RFLP analysis of the env region [*Calabro ML, et al., J Infect Dis 1993,* ***168:****1273-1277*]. Patients were HTLV-1 and HIV-1 negative and presented a high HTLV-2 proviral loads (ranging from 2.5 to 5.7% of the infected PBMCs). HTLV-2 proviral loads were determined by Real Time PCR amplification of the *pol* gene and were calculated as [(*pol* copy number/*albumin* copy number) x 10^5 PBMCs]. None of the patients examined was under antiviral therapy.
